# Intravenous arylsulfatase A in metachromatic leukodystrophy: a phase 1/2 study

**DOI:** 10.1002/acn3.51254

**Published:** 2020-12-17

**Authors:** Christine í Dali, Samuel Groeschel, Mihai Moldovan, Mohamed H. Farah, Ingeborg Krägeloh‐Mann, Margaret Wasilewski, Jing Li, Norman Barton, Christian Krarup

**Affiliations:** ^1^ Department of Clinical Genetics Rigshospitalet Copenhagen Denmark; ^2^ Department of Neuropediatrics University Children’s Hospital Tübingen Tübingen Germany; ^3^ Department of Clinical Neurophysiology Rigshospitalet Copenhagen Denmark; ^4^ Department of Neuroscience University of Copenhagen Copenhagen Denmark; ^5^ Department of Neurology Johns Hopkins School of Medicine Baltimore Maryland USA; ^6^ Shire (a member of the Takeda group of companies) Lexington Massachusetts USA; ^7^ Present address: Orphazyme A/S Copenhagen Denmark; ^8^ Present address: Summit Therapeutics PLC Cambridge Massachusetts USA

## Abstract

**Objective:**

Metachromatic leukodystrophy (MLD) is an autosomal recessive lysosomal storage disease caused by deficient activity of arylsulfatase A (ASA), resulting in severe motor and cognitive dysfunction. This phase 1/2 study evaluated the safety and efficacy of intravenous (IV) recombinant human ASA (rhASA; HGT‐1111, previously known as Metazym) in children with MLD.

**Methods:**

Thirteen children with MLD (symptom onset < 4 years of age) were enrolled in an open‐label, nonrandomized, dose‐escalation trial and received IV rhASA at 50, 100, or 200 U/kg body weight every 14 (± 4) days for 52 weeks (NCT00418561; NCT00633139). Eleven children continued to receive rhASA at 100 or 200 U/kg during a 24‐month extension period (NCT00681811). Outcome measures included safety observations, changes in motor and cognitive function, and changes in nerve conduction and morphometry.

**Results:**

There were no serious adverse events considered related to IV rhASA. Motor function and developmental testing scores declined during the study in all dose groups; no significant differences were observed between groups. Nerve conduction studies and morphometric analysis indicated that peripheral nerve pathology did not worsen during the study in any dose group.

**Interpretation:**

IV rhASA was generally well tolerated. There was no evidence of efficacy in preventing motor and cognitive deterioration, suggesting that IV rhASA may not cross the blood–brain barrier in therapeutic quantities. The relative stability of peripheral nerve function during the study indicates that rhASA may be beneficial if delivered to the appropriate target site and supports the development of rhASA for intrathecal administration in MLD.

## Introduction

Metachromatic leukodystrophy (MLD; OMIM 250100) is a rare, life‐limiting, autosomal recessive lysosomal storage disease (LSD) caused by deficient arylsulfatase A (ASA) activity. Progressive sulfatide accumulation in the central (CNS) and peripheral (PNS) nervous systems leads to progressive demyelination, accompanied by cognitive and motor function decline.^1^, ^2^, ^3^ MLD has been classified into three clinical forms corresponding to age of onset: late‐infantile, juvenile, and adult (onset at < 30 months, 2.5–16 years, and > 16 years, respectively).^1^, ^2^, ^3^, ^4^ The subtype depends largely on the inherited mutation in the arylsulfatase A (*ARSA*) gene and the resulting level of ASA activity, with more than 200 *ARSA* mutations identified.^5^

Late‐infantile MLD is the most frequent and severe form of MLD, accounting for approximately 50% of cases.^2^ Early manifestations generally include abnormal movement patterns and gait disturbances, such as instability when standing, followed by a rapid decline in motor function and cognition, with death typically occurring during childhood.^1^, ^2^, ^3^, ^6^, ^7^ For patients with juvenile and adult MLD, cognitive and behavioral symptoms are often observed first, followed by a more protracted decline in motor function.^6^, ^7^ These distinctions are not absolute, however, and disease severity may be considered a continuum, with particular overlap between late‐infantile and early‐juvenile presentation.^1^, ^8^

There are no approved therapies for MLD.^4^ Although hematopoietic stem cell transplantation has been reported to delay disease progression in some patients, efficacy is limited for patients with late‐infantile MLD and those who are symptomatic.^9^, ^10^ Alternative potential treatment approaches in development include lentiviral gene therapy^11^, ^12^ and enzyme replacement therapy (ERT). Intravenous (IV) ERT is known to stabilize or improve somatic manifestations of other LSDs, including mucopolysaccharidosis (MPS) types I, II, IVA, VI, and VII, acid lipase deficiency, and Fabry, Pompe, and Gaucher diseases.^13^, ^14^, ^15^, ^16^ The efficacy of IV ERT for the CNS symptoms of LSDs is limited by the inability of intravenously delivered proteins to cross the blood–brain barrier in therapeutic amounts.^17^, ^18^ However, studies using mouse models of MLD indicated that IV ERT improved CNS histopathology and motor and behavioral symptoms, warranting further investigation of this treatment approach for MLD.^19^, ^20^

In the present study, the safety and efficacy of IV ERT with recombinant human ASA (rhASA; HGT‐1111, previously known as Metazym) were evaluated in a phase 1/2 trial in children with MLD. Previous exploratory analyses of baseline data from patients enrolled in this trial provided evidence of disease burden in both the CNS and PNS.^21^, ^22^ *N*‐acetylaspartate (NAA), a marker of neuroaxonal integrity, was present at markedly reduced levels in the CNS of children with advanced disease, and NAA levels in brain white matter correlated with motor function assessed using the 88‐item Gross Motor Function Measure (GMFM‐88).^22^ PNS damage correlated with sural nerve and cerebrospinal fluid (CSF) (lyso)sulfatide levels, which were not markers for CNS damage.^21^ We present efficacy and safety data from children with MLD treated with IV rhASA in this phase 1/2 trial and extension. CNS and PNS efficacy data were obtained to assess the effect of IV rhASA on each.

## Methods

The objective of this phase 1/2 study was to assess the safety, efficacy, and pharmacokinetics of IV rhASA in children with MLD treated for two consecutive 26‐week periods (Study‐048; NCT00418561; NCT00633139). The objectives of the long‐term extension (Study‐049; NCT00681811) were to provide ongoing treatment with IV rhASA to patients who had completed Study‐048 and to assess disease progression and long‐term safety. All study‐related documents, including protocols, protocol amendments, and informed consent, were reviewed and approved by the Ethics Committees for Copenhagen and Frederiksberg Municipalities, Denmark. The studies conformed to the standards of conduct for clinical studies as detailed in the Declaration of Helsinki and the legal regulations in Denmark. International Conference on Harmonization guidelines for good clinical practices were followed. Each patient’s legal guardian(s) provided informed consent before study‐related activities were performed.

### Study Population

Thirteen children were enrolled; baseline characteristics of the study population have been described previously.^21^

Inclusion criteria were as follows: diagnosis of MLD established by ASA activity below 10 nmol/h per mg in leukocytes and elevated urine sulfatide levels; aged 12 months–6 years at screening, with symptom onset before 4 years of age; presence of residual, voluntary function, including cognitive (attention, executive, and visual) and motor (one upper or lower limb as a minimum) functions.^6^, ^7^, ^23^

Exclusion criteria were as follows: lack of voluntary function; severe bulbar signs (swallowing difficulty); spasticity sufficiently severe to hinder transportation to a treatment center; multiple sulfatase deficiency; major congenital abnormality; chromosomal abnormality; history of stem cell transplantation; clinically significant cardiovascular, hepatic, pulmonary, or renal disease; any other medical condition or serious intercurrent illness, or extenuating circumstance that, in the opinion of the investigator, would preclude study participation. Children were also ineligible if they had received any investigational product in the 30 days before enrollment, were enrolled in any other study involving clinical investigations, or were planning to initiate treatment for spasticity during the study.

### Study Design

#### Study‐048

Study‐048 was a 52‐week, single‐center, open‐label, nonrandomized, dose‐escalation, phase 1/2 trial conducted between January 2007 and September 2008. Patients from across Europe were included; the study was initiated at University Hospital Hvidovre, Copenhagen, Denmark, and clinical assessments took place either at this location or at Rigshospitalet, Copenhagen, Denmark.

Patients were allocated to one of the following three dose groups for infusion of IV rhASA every 14 (±4) days: first dose 25 U/kg body weight with subsequent 50 U/kg doses, 30‐min infusion; 100 U/kg, 30‐min infusion; 200 U/kg, 60‐min infusion. Attempts were made to ensure a similar spectrum of motor function across dose groups, but no formal stratification was applied. Dose levels were chosen based on preclinical pharmacology data for rhASA, with the selected minimum of 50 U/kg representing the lowest dose at which a beneficial effect was observed in mice,^19^ as well as an understanding of cellular uptake and pharmacological response for other ERTs. Doses were adjusted monthly to account for body weight changes.

Primary endpoints for the first 26‐week period of Study‐048 included the number of patients with treatment‐emergent adverse events (AEs), changes from baseline in GMFM‐88 and Mullen Scale of Early Learning (MSEL) scores, and change from baseline in CSF sulfatide levels. For the second 26‐week period of Study‐048, primary endpoints were the relative changes from baseline in GMFM‐88 and MSEL scores. Other outcomes assessed as secondary or exploratory endpoints included changes in nerve conduction, sural nerve biopsy findings, and magnetic resonance imaging (MRI) scores.

#### Study‐049

Children who completed Study‐048 were eligible to enroll in the extension (Study‐049), which started in February 2008 and was conducted at Rigshospitalet, Copenhagen, Denmark. Patients received IV rhASA 100 or 200 U/kg every 14 (± 4) days. Children who received 50 U/kg in Study‐048 were allocated 1:1 to receive either 100 or 200 U/kg in Study‐049. Children in the 100 and 200 U/kg groups in Study‐048 continued with these doses in Study‐049. Doses were adjusted every 6 weeks to account for body weight changes. During Study‐049, children not from Denmark were given the option to receive IV rhASA in their home country, subject to necessary approvals. The primary endpoint was number of days of exposure to IV rhASA, with secondary endpoints of CSF sulfatide levels, GMFM‐88 scores, and white matter metabolites (assessed using magnetic resonance spectroscopy [MRS]). Changes in nerve conduction were assessed as an exploratory outcome. Patients in Study‐049 were eligible to receive treatment until commercial availability of the product, discontinuation, or study termination by the sponsor, provided that no safety issues emerged. The study was terminated after 24 months owing to lack of efficacy and completed in October 2010.

### Efficacy Variables

Unless otherwise stated, efficacy assessments were performed at baseline, at 10, 26, and 52 weeks during Study‐048, and at 6‐monthly intervals during Study‐049.

#### Motor Function

Gross motor function was assessed by a physiotherapist who specialized in child development, using the GMFM‐88.^21^ This measures a child’s best abilities across several gross motor domains and is validated to describe gross motor skills and changing abilities over time in children with neurological disorders.^23^, ^24^, ^25^ The GMFM‐88 total score is the weighted normalized score across five dimensions, converted to a percentage of the maximum score.

#### Cognitive and Developmental Function

Early cognitive and developmental function was assessed by child psychologists, using the MSEL.^26^ This measures development across the domains of gross motor, visual reception, fine motor, receptive language, and expressive language. Scores were calculated as mean total scores across these dimensions.^26^, ^27^

#### Biomarkers in CSF and Sural Nerves

CSF sulfatide levels were assessed by thin‐layer chromatography and immunostaining with the Sulph I antisulfatide antibody, following a published methodology.^28^, ^29^ Sural nerve sulfatide and lysosulfatide levels (ng/mg dry weight) were assessed at baseline and 26 weeks, and were measured using high‐pressure liquid chromatography–mass spectrometry, as described previously.^21^

#### Albumin Index

CSF and serum albumin levels were assessed to determine the albumin index (a measure of blood–brain barrier integrity). The index was calculated as the ratio of CSF albumin (mg/dL) to serum albumin (g/dL). In healthy children, the ratio is typically below 9, depending on age.^30^, ^31^, ^32^, ^33^

#### Brain Imaging

Imaging was conducted using standard protocols, as described previously.^22^ MRIs were visually assessed on axial T2‐weighted and high‐resolution T1‐weighted sequences by four independent experts using a visual scoring system validated for MLD.^34^ Echo‐planar spectroscopy provided multi‐slice metabolic maps from MRS, and NAA levels were determined, as described previously.^22^ MRI score and NAA levels were assessed at baseline and 26 and 52 weeks during Study‐048; NAA levels were also assessed at 6‐monthly intervals during Study‐049. NAA is a marker of neuroaxonal density, with lower NAA levels corresponding to lower neuroaxonal density.^35^

#### Electrophysiological Assessments

Children were sedated and nerve conduction studies were performed in the median (sensory and motor fibers), fibular (motor fibers), and sural (sensory fibers) nerves, as described previously.^21^ Assessments of nerve conduction were performed at baseline, at 10, 26, and 52 weeks during Study‐048, and at 78 weeks in Study‐049. Somatosensory evoked potentials were recorded at the spine and scalp after electrical stimulation of tibial nerves at the medial malleolus and the median nerves at the wrist, as described previously.^21^

#### Nerve Morphometry

Light microscopic examinations were performed on biopsies of sural nerves obtained during Study‐048 at baseline and 26 weeks, as described previously.^21^ Myelin rings were traced on nerve cross sections and the areas were used to calculate myelin fiber diameters. The distribution of fiber diameters and fiber counts was calculated from the total endoneurial area.

### Safety

Safety parameters assessed included AEs, clinical laboratory assessments, vital signs, electrocardiogram evaluations, physical examinations, and antibody profiles.

AEs were summarized by system organ class and preferred term according to the Medical Dictionary for Regulatory Activities (MedDRA; version 8.2). An AE was defined as any untoward symptom or sign (including abnormal laboratory findings) experienced by patients from the time of consent until study completion, irrespective of whether it was considered related to IV rhASA. Patients were observed for at least 24 h after the first infusion and at least 2 h after subsequent infusions. Infusion‐related reactions (IRRs) were defined as AEs occurring from infusion initiation up to, and including, a 2‐h postinfusion observation period, which were assessed by the investigator as being related to IV rhASA administration.

Clinical laboratory assessments consisted of serum biochemistry and hematology. Vital signs comprised systolic and diastolic blood pressures, heart rate, body temperature (ear), and respiration rate. Electrocardiogram recordings were performed using a standard 12‐lead set‐up.

### Statistical Analysis

Relative changes from baseline in GMFM‐88 total score, MSEL score, and CSF sulfatide levels were calculated as a percentage change from baseline divided by the age difference between visits. Analyses of covariance (ANCOVA) were performed, with dose level as a fixed effect and baseline value as a covariate. Mean change from baseline was compared for each dose group and the corresponding 95% confidence interval constructed. However, the study was not powered for detecting statistical differences between dose groups.

*P‐*values for changes from baseline for electrophysiological parameters were obtained using nonparametric tests (Friedman test for related values and Kruskal–Wallis test for the whole material).

## Results

### Study Population

**Figure 1:**
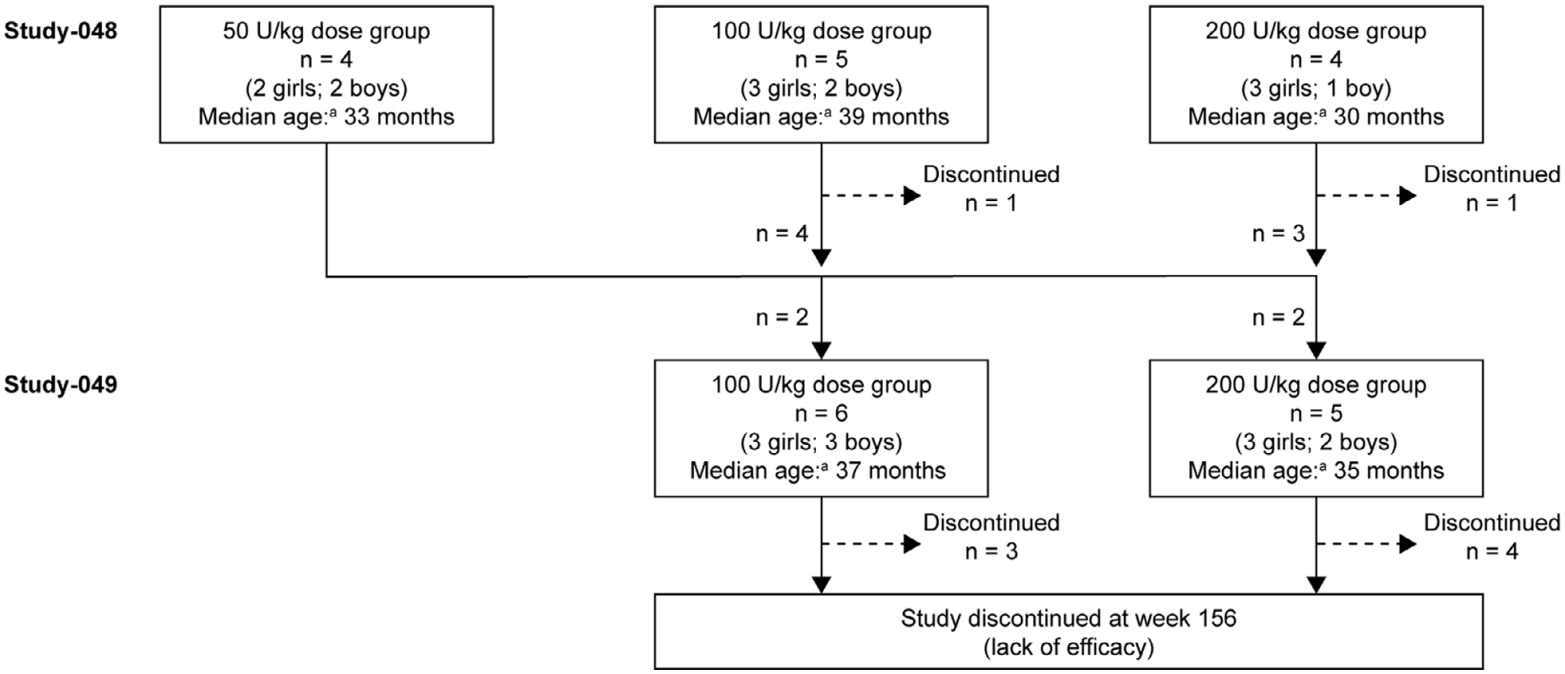
Study design and patient characteristics. ^a^Median age at baseline (the start of Study‐048).

Thirteen children (eight girls; five boys) with MLD and symptom onset before 4 years of age were enrolled in Study‐048 (Fig. 1). Median (range) age at baseline was 34.0 (24.0–59.0) months. Four children were allocated to rhASA 50 U/kg, five to 100 U/kg, and four to 200 U/kg.

Two children discontinued treatment in Study‐048: one (100 U/kg) at week 18 due to withdrawal of consent and one (200 U/kg) after week 30 due to lack of compliance. Eleven children completed Study‐048 and were enrolled in Study‐049: six received 100 U/kg and five received 200 U/kg (Fig. 1). Seven children discontinued treatment in Study‐049 (100 U/kg: one died from pneumonia, two withdrew consent; 200 U/kg: one due to an AE, one due to lack of compliance, two withdrew consent).

### Motor and Cognitive Assessments

#### GMFM‐88 Total Scores

Baseline mean GMFM‐88 total scores in Study‐048 were broadly similar in the three dose groups, and indicated that the disease was already well progressed at study start (Table 1). Decreases in mean GMFM‐88 total score, indicating declining motor function, were observed in all three dose groups from baseline at 52 weeks, and the relative decrease from baseline at week 52 was statistically significant in the 100 and 200 U/kg groups. There was no significant difference in the relative change from baseline between dose groups.

**Table 1:** Mean GMFM‐88 Total Scores and MSEL Scores by Study Week and Relative Change from Baseline to 52 and 78 Weeks

IV rhASA Dose, U/kg	Baseline (All Patients)	10 Weeks	26 Weeks	52 Weeks	Baseline (Patients in Study‐049)	78 Weeks	Relative Change from Baseline (95% CI) at 52 Weeks
Mean GMFM‐88 Total Score, %^1^							
50	23.2	22.0	8.2	5.7	−	−	–2.98 (–6.08; 0.12) *P* = 0.0574
100	17.9	14.7	12.3	6.0	25.9 (*n* = 6)	6.3 (*n* = 5)	–5.42 (–8.50; –2.34) *P = *0.0042
200	21.7	18.4	7.3	3.5	10.4 (*n* = 5)	3.4 (*n* = 5)	–5.28 (–8.91; –1.65) *P* = 0.0109
				Test for no difference between groups (52 weeks)			0.4013
Mean MSEL Total Score^2^							
50	11.9	15.6	9.6	4.4	−	−	–2.82 (–6.31; 0.66) *P* = 0.0948
100	14.0	10.8	8.9	7.8	14.5 (*n* = 6)	5.9 (*n* = 5)	–2.97 (–6.47; 0.54) *P* = 0.0840
200	11.8	9.8	7.8	2.0	7.9 (*n* = 5)	2.6 (*n* = 4)	–6.98 (–11.95; –2.01) *P* = 0.0139
				Test for no difference between groups (52 weeks)			0.2750

Relative changes from baseline were calculated as a percentage change from baseline divided by the age difference between visits.

CI, confidence interval; GMFM‐88, 88‐item Gross Motor Function Measure; IV, intravenous; MSEL, Mullen Scales of Early Learning; rhASA, recombinant human arylsulfatase A.

^1^ The GMFM‐88 total score is the weighted normalized score across five dimensions, converted to a percentage of the maximum score. Children aged 2–3 years (i.e., similar ages to this study population at baseline) and with normal motor abilities would be expected to achieve a total score above 80, and those aged 5 years or older with normal motor abilities would be expected to obtain a total score of 100.^36^

^2^ The MSEL total score is calculated as the mean total scores across five dimensions.

For the 11 patients who were enrolled in Study‐049, mean GMFM‐88 total scores continued to decline relative to baseline in both dose groups up to week 78 (i.e., week 26 of Study‐049), with no significant difference observed between groups (Table 1).

#### MSEL Total Scores

Mean MSEL total scores were similar across dose groups at baseline in Study‐048, and indicated that cognitive development was already substantially impaired at study start (Table 1). Further decreases were observed in all dose groups from baseline at week 52, and also at week 78 for those patients who continued into Study‐049.

Statistical analysis indicated that there was a significant relative decrease in the mean MSEL total score from baseline at week 52 in the 200 U/kg group. There was no significant difference between dose groups in the change in MSEL total score over time.

### Biomarkers

#### CSF Sulfatide Levels and Albumin Index

In each dose group, mean CSF sulfatide levels at baseline (Table 2) were, on average, over 20 times higher than the levels observed in healthy controls (< 50 nmol/L).^21^ In the 100 and 200 U/kg groups, mean levels decreased relative to baseline and remained lower than baseline in Study‐048 throughout the 52 weeks, whereas in the 50 U/kg group, mean levels increased relative to baseline (Table 2). At week 52, relative mean changes from baseline were not statistically significant in any of the dose groups, and no significant difference was observed between groups (*p* = 0.1363).

**Table 2:** Individual and Mean CSF Sulfatide Levels and Albumin Index at Baseline and 10, 26, and 52 Weeks

IV rhASA Dose, U/kg	Patient	Sulfatides (nmol/L) in CSF				Albumin Index^1^			
		Baseline	10 Weeks	26 Weeks	52 Weeks	Baseline	10 Weeks	26 Weeks	52 Weeks
50	Patient A	ND	1400	1180	1800	ND	23	54	37
	Patient B	200	350	550	550	5	5	10	8
	Patient C	750	550	875	750	28	14	21	15
	Patient D	550	950	2550	1550	25	28	56	34
	Mean	500	813	1289	1163	19	18	35	24
100	Patient E	1450	1300	1100	1500	31	26	27	28
	Patient F^2^	2300	1650	–	–	52	46	–	–
	Patient G	700	532	350	325	5	4	6	5
	Patient H	225	150	150	115	2	2	3	2
	Patient I	675	800	1025	970	13	14	16	15
	Mean	1070	886	656	728	21	18	13	13
200	Patient J	1200	675	575	800	35	17	22	15
	Patient K	2200	1000	725	700	45	31	29	22
	Patient L	1930	775	760	1000	33	37	29	27
	Patient M^3^	1150	ND	1100	–	20	ND	20	–
	Mean	1620	817	790	833	33	28	25	21

CSF, cerebrospinal fluid; IV, intravenous; ND, not determined; rhASA, recombinant human arylsulfatase A.

^1^ Albumin index was calculated as the ratio of CSF albumin (mg/dL) to serum albumin (g/dL).

^2^ Patient was discontinued from Study‐048 at week 18 due to withdrawal of consent.

^3^ Patient was discontinued from Study‐048 at week 30 due to lack of compliance.

The mean albumin index decreased from baseline to week 52 in the 100 and 200 U/kg groups, but not in the 50 U/kg group (Table 2). Albumin index values for individual patients showed that children with higher CSF sulfatide levels also tended to have higher albumin index values. Patients B, G, and H had low albumin index values during the study, and these same children also had lower than average CSF sulfatide levels.

#### Sural Nerve Sulfatide and Lysosulfatide Levels

**Figure 2:**
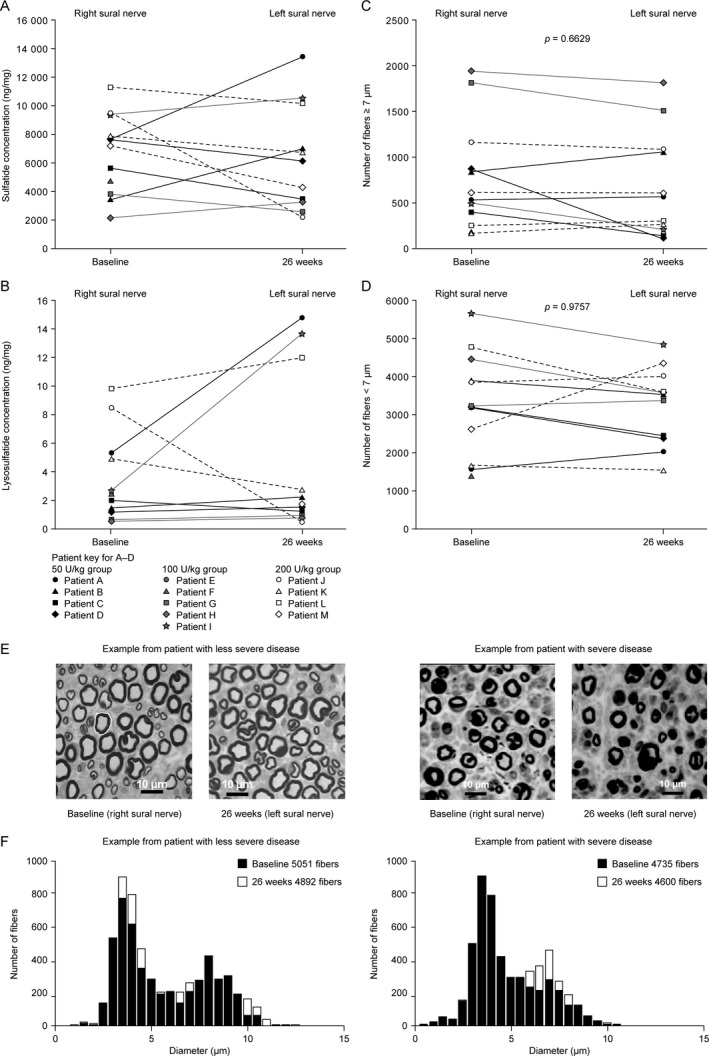
Sulfatide measurements, lysosulfatide measurements, and light microscopic measurements of the right sural nerve at baseline and the left sural nerve at 26 weeks. (A) Sulfatide concentrations in all patients with available data (*n* = 12 at baseline; *n* = 11 at 26 weeks). (B) Lysosulfatide concentrations in all patients with available data (*n* = 11 at baseline and 26 weeks). (C) Total number of large fibers> 7 µm in diameter (cross‐section of whole nerve) in all patients with available data (*n* = 12 at baseline; *n* = 11 at 26 weeks). (D) Total number of small fibers < 7 µm in diameter (cross‐section of whole nerve) in all patients with available data (*n* = 12 at baseline; *n* = 11 at 26 weeks). (E) Examples of transverse light microscopic sections of the sural nerves in two patients. (F) Fiber diameter distributions for sural nerves from the patients in (E). The external diameters of fibers were traced (as shown in E) and the diameter of the fiber was calculated from the area enclosed by the trace. The diameters were compared using paired nonparametric tests.

Available baseline values for sural nerve sulfatide and lysosulfatide levels (Fig. 2A and B; range: 2160–11 300 ng/mg sulfatide; 0.53–9.85 ng/mg lysosulfatide) were substantially higher than mean values observed in controls without MLD (935 ng/mg sulfatide; 0.14 ng/mg lysosulfatide).^21^ Sulfatide and lysosulfatide levels remained relatively stable from baseline to week 26 in the majority of patients, but there was also considerable interindividual variability, with large increases or decreases observed for some patients (Fig. 2A and B).

### Peripheral Nerve Assessments

#### Nerve Morphometry

Morphometric measurements of the sural nerves, obtained using light microscopy, showed no statistically significant changes from baseline to 26 weeks in the number of fibers ≥ 7 µm or < 7 µm in diameter (Fig. 2C to F).

#### Electrophysiological findings

**Figure 3:**
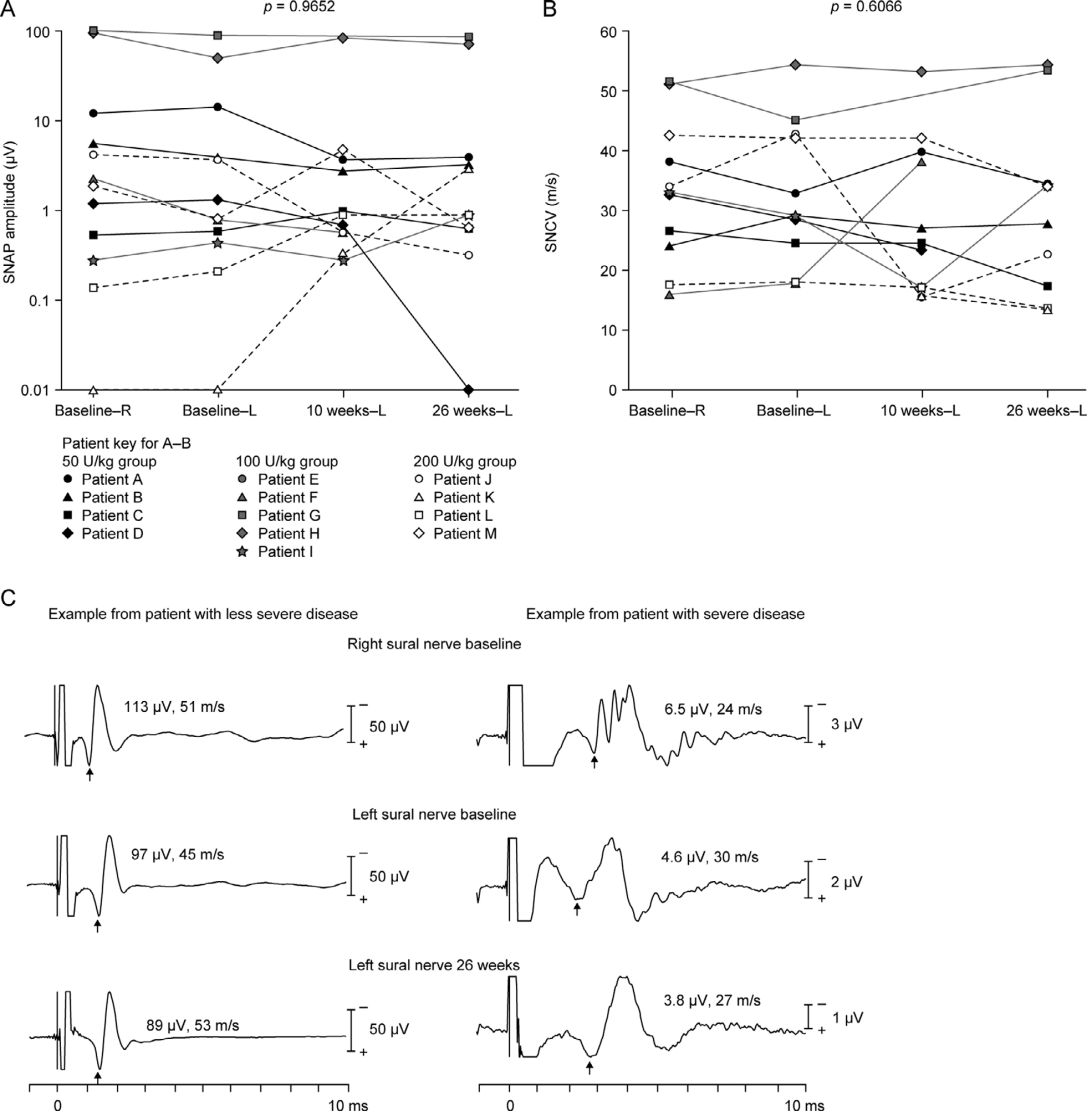
Sensory nerve conduction studies of the right and left sural nerves at baseline and the left sural nerve at 10 and 26 weeks. (A) SNAP amplitudes in all patients with available data (*n* = 12 at baseline; *n* = 11 at 10 weeks and 26 weeks). Values measured as zero are plotted at 0.01 to enable visualization on the logarithmic scale. (B) SNCVs in all patients with available data (*n* = 11 at baseline and 10 weeks; *n* = 10 at 26 weeks). (C) Example SNAP recordings in two patients, one with less severe disease and one with severe disease. The values above the traces refer to SNAP amplitudes (μV) and SNCVs (m/s). Comparisons of the parameters were carried out using nonparametric tests. L, left; R, right; SNAP, sensory nerve action potential; SNCV, sensory nerve conduction velocity.

**Figure 4:**
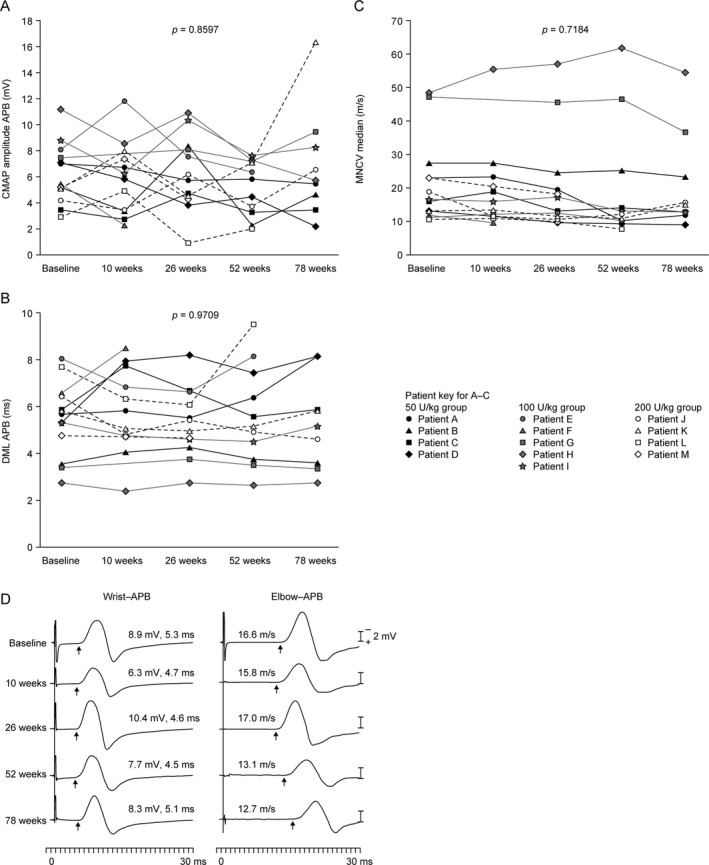
Motor nerve conduction studies of the right median nerve at baseline and 10, 26, 52, and 78 weeks. (A) CMAP amplitudes in the APB in all patients with available data (*n* = 13 at baseline; *n* = 12 at 10 weeks and 26 weeks; *n* = 11 at 52 weeks; *n* = 9 at 78 weeks). (B) DMLs in the APB in all patients with available data (*n* = 13 at baseline; *n* = 12 at 10 weeks and 26 weeks; *n* = 11 at 52 weeks; *n* = 9 at 78 weeks). (C) MNCVs of the median nerve in all patients with available data (*n* = 13 at baseline; *n* = 12 at 10 weeks and 26 weeks; *n* = 11 at 52 weeks; *n* = 9 at 78 weeks). (D) Example recordings of median nerve action potentials in an individual patient. The values above the traces refer to CMAP amplitudes (mV), DMLs (ms), and MNCVs (m/s). Comparisons of the parameters were carried out using nonparametric tests. APB, abductor pollicis brevis; CMAP, compound muscle action potential; DML, distal motor latency; MNCV, motor nerve conduction velocity.

There were no statistically significant changes in sensory nerve action potential amplitudes or sensory nerve conduction velocities at 26 weeks (Fig. 3). In addition, no statistically significant changes from baseline were observed in compound muscle action potential amplitudes, distal motor latencies, or motor nerve conduction velocities in the right median nerve (Fig. 4) or fibular nerves (data not shown). Notably, these assessments suggested that sensory and motor nerve conduction was normal at the study start and throughout the observation period in two children.^37^

Evoked potential recordings showed markedly prolonged cortical latencies when stimulating the median and tibial nerves, and there were no significant changes in latencies through the observation period (data not shown).

### Brain Imaging

#### MRI Total Scores

**Figure 5:**
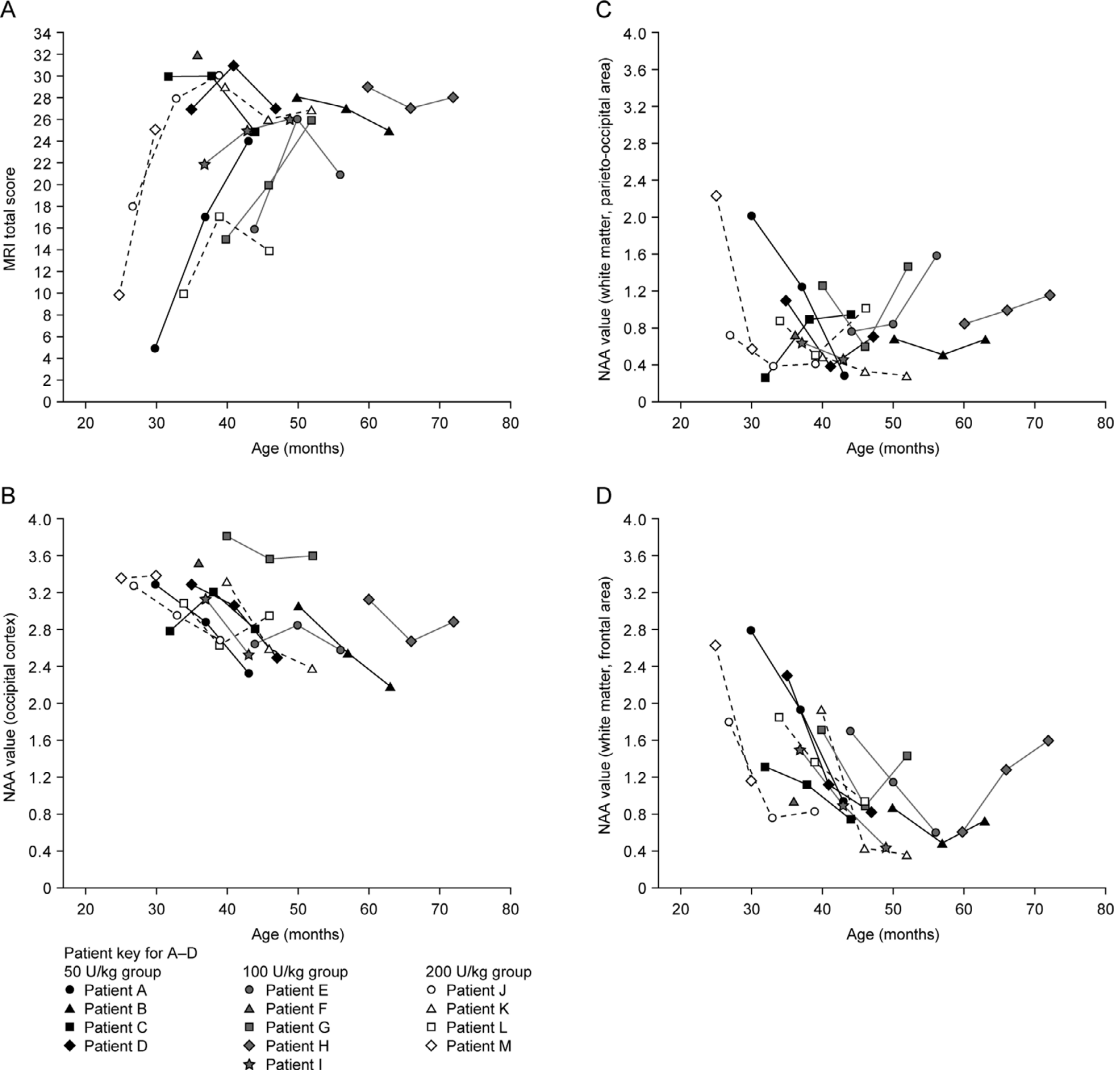
Individual MRI total scores and MRS‐derived NAA values from baseline to 52 weeks by patient age. (A) MRI total scores. (B) MRS‐derived NAA values from the occipital cortex. (C) MRS‐derived NAA values from white matter, parieto‐occipital area. (D) MRS‐derived NAA values from white matter, frontal area. IV, intravenous; MRI, magnetic resonance imaging; MRS, magnetic resonance spectroscopy; NAA, *N*‐acetylaspartate; rhASA, recombinant human arylsulfatase A.

MRI total scores during the study are shown for individual patients in Figure 5A. Mean (standard deviation) MRI total scores at baseline were 22.50 (11.73), 22.80 (7.60), and 16.75 (9.00) in the 50, 100, and 200 U/kg groups, respectively. Slight increases in mean score from baseline were observed in each group, indicating disease progression: mean changes at 52 weeks were 2.75 (11.03), 4.75 (4.92), and 4.67 (7.02), respectively.

#### MRS‐Derived NAA Values

In general, MRS‐derived NAA values from the occipital cortex and two white matter (parieto‐occipital and frontal) areas tended to decrease through the study, suggesting a potential reduction in neuronal integrity (Fig. 5B–D). However, increases were observed over time for some patients in the 100 U/kg group.

### Safety

Median (range) study drug exposure was 365 (121–379) days in Study‐048, with an additional 659 (85–945) days in Study‐049. The number of patients who experienced AEs is summarized in Table 3, and a summary of all treatment‐emergent AEs is provided in Tables S1 and S2.

**Table 3:** Summary of Patients Experiencing AEs

	IV rhASA Dose, U/kg									Total		
	50			100			200					
	N	(%)	E	N	(%)	E	N	(%)	E	N	(%)	E
Study‐048												
Experienced at least one AE	4	(100)	68	5	(100)	78	4	(100)	57	13	(100)	203
Experienced at least one drug‐related AE												
Probably related	2	(50)	15	2	(40)	17	3	(75)	12	7	(54)	44
Definitely related	2	(50)	2	1	(20)	1	0	(0)	−	3	(23)	3
Experienced at least one SAE	2	(50)	3	2	(40)	4	3	(75)	10	7	(54)	17
Discontinuation due to AE^1^	0	(0)	−	0	(0)	−	0	(0)	−	0	(0)	−
Deaths	0	(0)	−	0	(0)	−	0	(0)	−	0	(0)	−
Study‐049												
Experienced at least one AE	NA	NA	NA	6	(100)	146	5	(100)	120	11	(100)	266
Experienced at least one drug‐related AE	NA	NA	NA	3	(50)	53	3	(60)	38	6	(55)	91
Experienced at least one SAE	NA	NA	NA	2	(33)	7	4	(80)	10	6	(55)	17
Discontinuation due to AE^2^	NA	NA	NA	1	(17)	−	1	(20)	−	2	(18)	−
Deaths	NA	NA	NA	1	(17)	−	0	(0)	−	1	(9)	−

AE, adverse event; E, number of events; IV, intravenous; N, number of patients; NA, not applicable; rhASA, recombinant human arylsulfatase A; SAE, serious adverse event.

^1^ No patients in Study‐048 discontinued due to an AE. Two patients did not complete the study: one discontinued at week 18 due to withdrawal of consent, and one was withdrawn by the investigator after week 30 due to lack of compliance.

^2^ Two patients in Study‐049 discontinued due to AEs. One patient was discontinued by the investigator for bronchospasm, rash, and urticaria AEs, which were all considered severe and treatment‐related. The second patient died from pneumonia; this was classed as an AE leading to discontinuation but was not considered by the investigator to be related to treatment with the study drug.

All patients in Study‐048 experienced one or more AEs. Of 203 AEs in Study‐048, 47 (23%) were considered to be treatment‐related, of which 45 were IRRs (27 moderate, 18 mild). In total, 17 serious AEs (SAEs) were reported, none of which was considered related to treatment. Two patients developed drug‐specific antibodies. In Study‐048, there were no discontinuations due to AEs and no deaths.

In Study‐049, a total of 266 AEs were reported in 11 patients. Of these, 91 were considered by the investigator as related to treatment with study drug (all IRRs). In total, 6 patients (54.5%) experienced 17 SAEs, none of which was considered related to treatment. One patient died during Study‐049. The patient suffered a severe chest infection resulting in death 39 weeks after study entry; the event was reported by the investigator as severe but not related to treatment with the study drug. One patient was discontinued for AEs of bronchospasm, rash, and urticaria, which were all considered severe and treatment‐related.

Clinical laboratory assessments, vital signs, and electrocardiogram recordings in Study‐048 and Study‐049 did not raise any safety concerns.

## Discussion

This phase 1/2 trial and its extension assessed the safety and efficacy of IV rhASA in children with MLD. IV rhASA was generally well tolerated and no SAEs or deaths related to treatment were reported; one patient discontinued due to a treatment‐related AE. Patients in this trial already had advanced MLD at enrollment, with severely impaired gross motor abilities (mean baseline GMFM‐88 total scores below 25% in all dose groups; similarly aged children with normal motor abilities would be expected to score > 80%).^36^ There was a clear decline in motor function at 52 and 78 weeks, indicating continued disease progression. Mean MSEL total scores (as a measure of cognitive function) were also low at baseline compared with a normative population,^26^ and declined during the study in all dose groups in parallel with motor function, indicating worsening developmental impairment.

Peripheral nerve function, assessed by electrophysiology and morphometry as reported here and in a previous baseline analysis,^21^ was abnormal at baseline in most children, though median nerve function did appear to be preserved in some cases by comparison to a reference population.^37^ Peripheral nerve function did not appear to worsen during the study, suggesting that treatment with IV rhASA may have protected the children in this trial against further disease progression in the PNS.

For some patients, further deterioration of peripheral nerve function may not have been possible due to the potential floor effect of severe peripheral nerve damage at baseline. However, most children had relatively intact median nerve function at baseline, and two had relatively high conduction across all nerves, which remained stable during the trial, suggesting that not all patients were at the floor of response. Despite their preserved PNS function, these two children still developed gross motor dysfunction, which is a core feature of MLD.^21^ While it is possible that this stability of peripheral nerve function may reflect normal variation in disease course, these observations may also suggest that treatment with IV rhASA‐stabilized disease progression in the PNS, but was unable to halt the decline in gross motor function.

Overall, there was a clear discordance observed between the ongoing deterioration in gross motor function, which dominates the general symptomatic picture in children with early‐onset MLD,^6^ and the stability of peripheral nerve pathology. This may suggest a primary contribution of CNS pathology to motor deterioration in MLD, which is further supported by brain NAA and MRI data that indicate declining neuronal integrity over the course of the study. This is generally in line with previous findings that CNS pathology, as assessed by demyelination load, but not PNS pathology, as assessed by nerve conduction velocity, correlates with a decline in motor function in MLD.^38^ However, some case reports of children with MLD have suggested that in some patients with initial abnormalities in motor function, peripheral neuropathy may precede apparent CNS pathology.^39^ It therefore seems likely that both CNS and PNS damage may contribute to impaired motor function in MLD, and that it may be necessary for degradation of sulfatides to reach a critical threshold in both the CNS and PNS to achieve improvements in gross motor function. Further exploration of the correlations between the clinical and laboratory parameters investigated in this study are planned to better understand the outcomes of treatment with IV rhASA in the context of disease pathology and progression in the CNS and PNS.

Biomarker measurements were obtained as an exploratory measure of disease severity and potential damage to the blood–brain barrier. Previous research has shown that increased blood–brain barrier leakage and cognitive dysfunction are correlated, and increased levels of albumin in the CSF indicate blood–brain barrier leakage and brain dysfunction.^40^ In the current study, albumin index values were above normal in most children, indicating that blood–brain barrier integrity was impaired to some extent. It could be speculated that this increased barrier permeability enhances the potential for IV rhASA to reach the brain; however, the trends in this study were inconclusive, with no clear evidence that higher albumin indices led to increased degradation of CSF sulfatides. Although findings in a mouse model suggested a potential for IV rhASA to reach the CNS,^19^ it is important to consider species‐ and age‐specific variability in the function of the blood–brain barrier;^40^ in particular, a larger brain size may limit diffusibility in humans.^41^ There was a tendency for higher albumin index values in those patients with higher CSF sulfatide levels, which may suggest that these are both markers of disease severity. In line with this suggestion, three patients with the lowest albumin indices all had relatively preserved motor function at baseline (i.e., able to walk, sit, or grab); however, this correlation is not absolute, as other patients with higher albumin indices were also able to walk at baseline.^21^ Further research is required to understand the extent to which CSF sulfatide is a marker of disease severity, as a previous study did not identify any correlation between CSF sulfatide levels and GMFM‐88 score.^21^

The natural history of MLD in terms of electrophysiological and morphometric parameters assessed in the current trial remains unknown, and the fact that it was not possible to include a comparator placebo group makes it challenging to understand how nerve function would have changed without treatment. The inclusion of children with symptom onset up to the age of 4 years is likely to have contributed to some degree of heterogeneity within the study population, as this may encompass some children with late‐infantile and others with early‐juvenile MLD. The conclusions that can be drawn regarding treatment efficacy are also limited by the fact that this was a small, dose‐ranging study designed to assess safety and preliminary efficacy. Preclinical studies in mouse models of MLD found that improvements in nerve function following ERT were most pronounced in mice at an earlier stage of disease;^19^, ^20^ however, the small number of patients in the current study meant that it was not possible to evaluate correlations between age or disease severity at enrollment and response to treatment. The severity of disease at baseline and rapid progression may also have impacted the ability to measure change in clinical parameters in this study. In addition, MRS‐derived NAA data were not available for all patients, thus allowing only descriptive analyses. These observations suggest that patients with MLD should be enrolled in clinical trials before the disease has progressed beyond the point at which a clinically meaningful effect is likely to be observed.

Overall, the results indicated that treatment with IV rhASA may be associated with some slowing of disease progression in the PNS of children with MLD but is unable to reach the CNS in adequate amounts to address the predominant CNS component of the disease. The challenge of addressing CNS changes with ERT is also seen with mucopolysaccharidosis II, an LSD for which IV idursulfase improves somatic signs and symptoms of the disease, but does not cross the blood–brain barrier in therapeutic quantities and cannot mitigate CNS aspects of the disease.^17^

If symptoms of childhood‐onset MLD primarily reflect progression of CNS demyelination, as indicated by the current data, then restoring ASA function in the CNS, potentially via intrathecal administration of rhASA, might be effective in slowing disease progression.^42^ This suggestion is supported by findings from a preclinical study in a mouse model of MLD, in which delivery of IV rhASA directly into the CSF via intracerebroventricular administration corrected ataxic gait.^43^ Given the rapid deterioration following onset of late‐infantile MLD in particular, initiation of treatment early in the disease course is likely to be most effective. In addition, because therapeutics delivered intrathecally may also enter the bloodstream via CSF reabsorption,^44^ there is the possibility of a therapeutic effect from both central and peripheral ASA exposure with intrathecal rhASA administration. Results from a phase 1/2 trial of intrathecally delivered rhASA (NCT01510028) demonstrated potential benefit with this treatment in a subset of children with MLD.^45^ Based on these data, a phase 2b trial has been initiated with the primary aim of assessing efficacy and safety of intrathecal rhASA in symptomatic patients with late‐infantile MLD (NCT03771898). In the future, there may also be potential to improve rhASA activity further using protein engineering.^46^

In conclusion, although peripheral nerve function did not worsen in children with MLD treated with IV rhASA in this study, gross motor function continued to decline, and the trial was ultimately discontinued owing to lack of efficacy. At baseline, median motor nerve function was less severely affected than fibular motor nerve function, and a lack of progression in median nerve function provides potential evidence of a therapeutic effect of rhASA in the PNS. Improving delivery of rhASA to the brain via intrathecal administration may extend this protection to the CNS and help to mitigate the decline in gross motor function. The findings from this study therefore support further evaluation of intrathecally administered rhASA as the approach to treatment in children with MLD.

## Authors’ Contributions

**CíD:** Design of the study, acquisition and analysis of data as a Principal Investigator, and drafting the manuscript. **SG, MM, MHF, IKM, MW, JL:** Analysis of data and drafting the manuscript. **NB:** Conception and design of the study, analysis of data, and drafting the manuscript. **CK:** Design of the study, acquisition and analysis of data, and drafting the manuscript.

## Conflicts of Interest

**CíD** reports personal fees from University Hospital Copenhagen Rigshospitalet during the conduct of the study. **MM** has nothing to disclose. **MHF** has nothing to disclose. **IKM** reports grants from Shire (a Takeda company) during the conduct of the study. **MW** reports fees for consulting services from ID Remedies LLC. In addition, Dr Wasilewski has a patent pending**:** Wasilewski, Margaret, Wijatyk, Anna; c/o Shire Human Genetic Therapies, Inc., 300 Shire Way, Lexington, MA 02421 (US) PCT/US20 17/0 18440, Methods and Compositions for CNS Delivery of Arylsulfatase A. 17 February 2017. She was also an employee of Shire (a Takeda company) from July 2015 to August 2018 and is a Takeda stock owner. **JL** is an employee of Shire (a Takeda company). **NB** is an employee of Shire (a Takeda company). **SG** reports an institutional research grant from Shire (a Takeda company) outside of the submitted work. He serves as an advisor for trials in metachromatic leukodystrophy (Shire [a Takeda company], Orchard, Bioclinica, Homology Medicine) but receives no personal payment related to this role. **CK** reports grants and personal fees from Shire (a Takeda company) and grants from Danish Medical Research during the conduct of the study.

## Supporting information


**Table S1**. Incidence of Treatment‐Emergent Adverse Events in Study‐048 by System Organ Class, Preferred Term and Treatment Group

**Table S2**. Incidence of Treatment‐Emergent Adverse Events in Study‐049 by System Organ Class, Preferred Term and Treatment Group

## Data Availability

Shire (a Takeda company) does not plan to share data supporting the results reported in this article as there is a reasonable likelihood that individual patients could be re‐identified (due to the limited number of study sites/participants).
