# Supplementary material for: Intravenous arylsulfatase A in metachromatic leukodystrophy: a phase 1/2 study
Source: Ann Clin Transl Neurol. 2020 Dec 17;8(1):66–80. doi: 10.1002/acn3.51254 (PMC7818087; doi:10.1002/acn3.51254)
Supplement: Supplementary file 1 — Table S1. Incidence of Treatment‐Emergent Adverse Events in Study‐048 by System Organ Class, Preferred Term and Treatment Group [file ACN3-8-66-s001.docx]

**SUPPLEMENTARY TABLE 1.** **Incidence of Treatment-Emergent Adverse Events in Study-048 by System Organ Class, Preferred Term and Treatment Group**

| **System Organ Class** | **IV rhASA Dose, U/kg** | | | | | | **All** | |
| --- | --- | --- | --- | --- | --- | --- | --- | --- |
| **Preferred Term** | **50** | | **100** | | **200** | |  | |
|  | **Patients (n = 4) n (%)^a^** | **Events n^b^** | **Patients (n = 5) n (%)^a^** | **Events n^b^** | **Patients (n = 4) n (%)^a^** | **Events n^b^** | **Patients (n = 13) n (%)^a^** | **Events n^b^** |
| Nervous system disorders | 4 (100) | 23 | 5 (100) | 22 | 4 (100) | 11 | 13 (100) | 56 |
| Muscle spasticity | 4 (100) | 9 | 4 (80) | 11 | 1 (25) | 1 | 9 (69) | 21 |
| Hypotonia | 2 (50) | 7 | 4 (80) | 6 | 3 (75) | 6 | 9 (69) | 19 |
| Cognitive disorder | 2 (50) | 5 | 2 (40) | 3 | 1 (25) | 1 | 5 (38) | 9 |
| Speech disorder developmental | 1 (25) | 2 | 2 (40) | 2 | 0 | 0 | 3 (23) | 4 |
| Convulsion | 0 | 0 | 0 | 0 | 2 (50) | 2 | 2 (15) | 2 |
| Mutism | 0 | 0 | 0 | 0 | 1 (25) | 1 | 1 (8) | 1 |
| General disorders and administration-site conditions | 4 (100) | 21 | 3 (60) | 23 | 4 (100) | 17 | 11 (85) | 61 |
| Pyrexia | 3 (75) | 6 | 3 (60) | 4 | 3 (75) | 5 | 9 (69) | 15 |
| Infusion-related reaction | 2 (50) | 15 | 2 (40) | 18 | 3 (75) | 12 | 7 (54) | 45 |
| Discomfort | 0 | 0 | 1 (20) | 1 | 0 | 0 | 1 (8) | 1 |
| Infections and infestations | 4 (100) | 14 | 3 (60) | 7 | 4 (100) | 9 | 11 (85) | 30 |
| Gastroenteritis | 3 (75) | 3 | 2 (40) | 2 | 1 (25) | 1 | 6 (46) | 6 |
| Pneumonia | 1 (25) | 1 | 1 (20) | 1 | 3 (75) | 4 | 5 (38) | 6 |
| Nasopharyngitis | 1 (25) | 1 | 1 (20) | 1 | 1 (25) | 1 | 3 (23) | 3 |
| Bronchitis acute | 2 (50) | 2 | 0 | 0 | 0 | 0 | 2 (15) | 2 |
| Tonsillitis | 1 (25) | 1 | 0 | 0 | 1 (25) | 1 | 2 (15) | 2 |
| Otitis media | 1 (25) | 2 | 0 | 0 | 0 | 0 | 1 (8) | 2 |
| Bronchitis | 0 | 0 | 0 | 0 | 1 (25) | 1 | 1 (8) | 1 |
| Acute tonsillitis | 1 (25) | 1 | 0 | 0 | 0 | 0 | 1 (8) | 1 |
| Varicella | 0 | 0 | 1 (20) | 1 | 0 | 0 | 1 (8) | 1 |
| Urinary tract infection | 1 (25) | 1 | 0 | 0 | 0 | 0 | 1 (8) | 1 |
| Postoperative infection | 0 | 0 | 0 | 0 | 1 (25) | 1 | 1 (8) | 1 |
| Viral infection | 1 (25) | 1 | 0 | 0 | 0 | 0 | 1 (8) | 1 |
| Influenza | 0 | 0 | 1 (20) | 1 | 0 | 0 | 1 (8) | 1 |
| Herpangina | 1 (25) | 1 | 0 | 0 | 0 | 0 | 1 (8) | 1 |
| Enterobiasis | 0 | 0 | 1 (20) | 1 | 0 | 0 | 1 (8) | 1 |
| Investigations | 3 (75) | 3 | 3 (60) | 7 | 2 (50) | 3 | 8 (62) | 13 |
| Hemoglobin decreased | 0 | 0 | 1 (20) | 1 | 1 (25) | 2 | 2 (15) | 3 |
| General physical condition abnormal | 0 | 0 | 1 (20) | 1 | 1 (25) | 1 | 2 (15) | 2 |
| Drug-specific antibody present | 2 (50) | 2 | 0 | 0 | 0 | 0 | 2 (15) | 2 |
| Blood iron increased | 0 | 0 | 1 (20) | 1 | 0 | 0 | 1 (8) | 1 |
| Blood iron decreased | 1 (25) | 1 | 0 | 0 | 0 | 0 | 1 (8) | 1 |
| Blood alkaline phosphatase increased | 0 | 0 | 1 (20) | 1 | 0 | 0 | 1 (8) | 1 |
| Alanine aminotransferase increased | 0 | 0 | 1 (20) | 1 | 0 | 0 | 1 (8) | 1 |
| Neutrophil count increased | 0 | 0 | 1 (20) | 1 | 0 | 0 | 1 (8) | 1 |
| White blood cell count decreased | 0 | 0 | 1 (20) | 1 | 0 | 0 | 1 (8) | 1 |
| Gastrointestinal disorders | 1 (25) | 1 | 3 (60) | 8 | 3 (75) | 6 | 7 (54) | 15 |
| Vomiting | 0 | 0 | 3 (60) | 4 | 2 (50) | 4 | 5 (38) | 8 |
| Diarrhea | 0 | 0 | 1 (20) | 1 | 1 (25) | 1 | 2 (15) | 2 |
| Abdominal pain upper | 0 | 0 | 1 (20) | 1 | 0 | 0 | 1 (8) | 1 |
| Nausea | 0 | 0 | 1 (20) | 1 | 0 | 0 | 1 (8) | 1 |
| Peritonitis | 0 | 0 | 0 | 0 | 1 (25) | 1 | 1 (8) | 1 |
| Flatulence | 0 | 0 | 1 (20) | 1 | 0 | 0 | 1 (8) | 1 |
| Constipation | 1 (25) | 1 | 0 | 0 | 0 | 0 | 1 (8) | 1 |
| Respiratory, thoracic and mediastinal disorders | 2 (50) | 3 | 3 (60) | 3 | 1 (25) | 2 | 6 (46) | 8 |
| Pharyngolaryngeal pain | 2 (50) | 2 | 1 (20) | 1 | 0 | 0 | 3 (23) | 3 |
| Cough | 0 | 0 | 1 (20) | 1 | 1 (25) | 1 | 2 (15) | 2 |
| Pneumonia aspiration | 0 | 0 | 1 (20) | 1 | 1 (25) | 1 | 2 (15) | 2 |
| Pharyngeal edema | 1 (25) | 1 | 0 | 0 | 0 | 0 | 1 (8) | 1 |
| Metabolism and nutrition disorders | 1 (25) | 1 | 2 (40) | 2 | 2 (50) | 3 | 5 (38) | 6 |
| Malnutrition | 1 (25) | 1 | 2 (40) | 2 | 2 (50) | 3 | 5 (38) | 6 |
| Injury, poisoning and procedural complications | 1 (25) | 1 | 1 (20) | 1 | 3 (75) | 3 | 5 (38) | 5 |
| Procedural pain | 1 (25) | 1 | 0 | 0 | 1 (25) | 1 | 2 (15) | 2 |
| Postprocedural vomiting | 0 | 0 | 0 | 0 | 1 (25) | 1 | 1 (8) | 1 |
| Medical device complication | 0 | 0 | 0 | 0 | 1 (25) | 1 | 1 (8) | 1 |
| Feeding tube complication | 0 | 0 | 1 (20) | 1 | 0 | 0 | 1 (8) | 1 |
| Musculoskeletal and connective tissue disorders | 1 (25) | 1 | 3 (60) | 3 | 1 (25) | 1 | 5 (38) | 5 |
| Muscle spasms | 1 (25) | 1 | 3 (60) | 3 | 1 (25) | 1 | 5 (38) | 5 |
| Psychiatric disorders | 0 | 0 | 1 (20) | 1 | 1 (25) | 1 | 2 (15) | 2 |
| Sleep disorder | 0 | 0 | 0 | 0 | 1 (25) | 1 | 1 (8) | 1 |
| Depression | 0 | 0 | 1 (20) | 1 | 0 | 0 | 1 (8) | 1 |
| Congenital, familial and genetic disorders | 0 | 0 | 1 (20) | 1 | 0 | 0 | 1 (8) | 1 |
| Leukodystrophy | 0 | 0 | 1 (20) | 1 | 0 | 0 | 1 (8) | 1 |
| Eye disorders | 0 | 0 | 0 | 0 | 1 (25) | 1 | 1 (8) | 1 |
| Blindness | 0 | 0 | 0 | 0 | 1 (25) | 1 | 1 (8) | 1 |

^a^Number (%) of patients in treatment group having the event.

^b^Number of events. Note that a patient may experience more than one event.

IV = intravenous; rhASA = recombinant human arylsulfatase A.
