# Supplementary material for: Intravenous arylsulfatase A in metachromatic leukodystrophy: a phase 1/2 study
Source: Ann Clin Transl Neurol. 2020 Dec 17;8(1):66–80. doi: 10.1002/acn3.51254 (PMC7818087; doi:10.1002/acn3.51254)
Supplement: Supplementary file 2 — Table S2. Incidence of Treatment‐Emergent Adverse Events in Study‐049 by System Organ Class, Preferred Term and Treatment Group [file ACN3-8-66-s002.docx]

**SUPPLEMENTARY TABLE 2. Incidence of Treatment-Emergent Adverse Events in Study-049 by System Organ Class, Preferred Term and Treatment Group**

| **System Organ Class** | **IV rhASA Dose, U/kg** | | | | **All** | |
| --- | --- | --- | --- | --- | --- | --- |
| **Preferred Term** | **100** | | **200** | |  | |
|  | **Patients (n = 6) n (%)^a^** | **Events n^b^** | **Patients (n = 5) n (%)^a^** | **Events n^b^** | **Patients (n = 11) n (%)^a^** | **Events n^b^** |
| Infections and infestations | 6 (100) | 38 | 5 (100) | 31 | 11 (100) | 69 |
| Bronchitis | 3 (50) | 4 | 3 (60) | 4 | 6 (55) | 8 |
| Nasopharyngitis | 4 (67) | 12 | 1 (20) | 2 | 5 (45) | 14 |
| Pharyngitis | 2 (33) | 5 | 2 (40) | 4 | 4 (36) | 9 |
| Bronchitis acute | 1 (17) | 1 | 2 (40) | 3 | 3 (27) | 4 |
| Eye infection | 1 (17) | 1 | 2 (40) | 3 | 3 (27) | 4 |
| Pneumonia | 1 (17) | 1 | 2 (40) | 3 | 3 (27) | 4 |
| Gastroenteritis | 1 (17) | 1 | 1 (20) | 2 | 2 (18) | 3 |
| Influenza | 1 (17) | 2 | 1 (20) | 2 | 2 (18) | 4 |
| Laryngitis | 1 (17) | 1 | 1 (20) | 1 | 2 (18) | 2 |
| Varicella | 1 (17) | 1 | 1 (20) | 1 | 2 (18) | 2 |
| Catheter bacteremia | 1 (17) | 2 | 0 | 0 | 1 (9) | 2 |
| Ear infection | 1 (17) | 1 | 0 | 0 | 1 (9) | 1 |
| Herpes simplex | 0 | 0 | 1 (20) | 1 | 1 (9) | 1 |
| Infection | 0 | 0 | 1 (20) | 1 | 1 (9) | 1 |
| Lower respiratory tract infection | 1 (17) | 2 | 0 | 0 | 1 (9) | 2 |
| Lung infection | 1 (17) | 1 | 0 | 0 | 1 (9) | 1 |
| Oral candidiasis | 0 | 0 | 1 (20) | 1 | 1 (9) | 1 |
| Rhinitis | 1 (17) | 1 | 0 | 0 | 1 (9) | 1 |
| Scarlet fever | 0 | 0 | 1 (20) | 1 | 1 (9) | 1 |
| Sinusitis | 0 | 0 | 1 (20) | 1 | 1 (9) | 1 |
| Tooth infection | 1 (17) | 1 | 0 | 0 | 1 (9) | 1 |
| Urinary tract infection | 0 | 0 | 1 (20) | 1 | 1 (9) | 1 |
| Viral pharyngitis | 1 (17) | 1 | 0 | 0 | 1 (9) | 1 |
| Metabolism and nutrition disorders | 0 | 0 | 2 (40) | 3 | 2 (18) | 3 |
| Dehydration | 0 | 0 | 1 (20) | 1 | 1 (9) | 1 |
| Malnutrition | 0 | 0 | 1 (20) | 2 | 1 (9) | 2 |
| Psychiatric disorders | 1 (17) | 1 | 2 (40) | 2 | 3 (27) | 3 |
| Sleep disorder | 0 | 0 | 2 (40) | 2 | 2 (18) | 2 |
| Mental status changes | 1 (17) | 1 | 0 | 0 | 1 (9) | 1 |
| Nervous system disorders | 4 (67) | 10 | 4 (80) | 16 | 8 (73) | 26 |
| Muscle spasticity | 2 (33) | 5 | 2 (40) | 3 | 4 (36) | 8 |
| Epilepsy | 1 (17) | 1 | 2 (40) | 6 | 3 (27) | 7 |
| Dystonia | 1 (17) | 1 | 1 (20) | 2 | 2 (18) | 3 |
| Convulsion | 0 | 0 | 1 (20) | 4 | 1 (9) | 4 |
| Depressed level of consciousness | 1 (17) | 1 | 0 | 0 | 1 (9) | 1 |
| Febrile convulsion | 1 (17) | 1 | 0 | 0 | 1 (9) | 1 |
| Hypotonia | 1 (17) | 1 | 0 | 0 | 1 (9) | 1 |
| Syncope | 0 | 0 | 1 (20) | 1 | 1 (9) | 1 |
| Eye disorders | 3 (50) | 4 | 0 | 0 | 3 (27) | 4 |
| Eyelid edema | 2 (33) | 2 | 0 | 0 | 2 (18) | 2 |
| Conjunctival edema | 1 (17) | 1 | 0 | 0 | 1 (9) | 1 |
| Visual acuity reduced | 1 (17) | 1 | 0 | 0 | 1 (9) | 1 |
| Ear and labyrinth disorders | 1 (17) | 1 | 0 | 0 | 1 (9) | 1 |
| Ear pain | 1 (17) | 1 | 0 | 0 | 1 (9) | 1 |
| Vascular disorders | 1 (17) | 1 | 3 (60) | 9 | 4 (36) | 10 |
| Flushing | 1 (17) | 1 | 3 (60) | 8 | 4 (36) | 9 |
| Pallor | 0 | 0 | 1 (20) | 1 | 1 (9) | 1 |
| Respiratory, thoracic and mediastinal disorders | 3 (50) | 7 | 4 (80) | 7 | 7 (64) | 14 |
| Cough | 3 (50) | 5 | 0 | 0 | 3 (27) | 5 |
| Pharyngolaryngeal pain | 1 (17) | 1 | 2 (40) | 2 | 3 (27) | 3 |
| Bronchospasm | 0 | 0 | 2 (40) | 2 | 2 (18) | 2 |
| Asthma | 0 | 0 | 1 (20) | 1 | 1 (9) | 1 |
| Epistaxis | 1 (17) | 1 | 0 | 0 | 1 (9) | 1 |
| Increased bronchial secretion | 0 | 0 | 1 (20) | 1 | 1 (9) | 1 |
| Respiratory distress | 0 | 0 | 1 (20) | 1 | 1 (9) | 1 |
| Gastrointestinal disorders | 6 (100) | 35 | 5 (100) | 17 | 11 (100) | 52 |
| Nausea | 3 (50) | 9 | 3 (60) | 3 | 6 (55) | 12 |
| Vomiting | 3 (50) | 15 | 3 (60) | 10 | 6 (55) | 25 |
| Constipation | 1 (17) | 1 | 2 (40) | 2 | 3 (27) | 3 |
| Gastritis | 2 (33) | 2 | 1 (20) | 1 | 3 (27) | 3 |
| Diarrhea | 2 (33) | 3 | 0 | 0 | 2 (18) | 3 |
| Gastroesophageal reflux disease | 1 (17) | 2 | 1 (20) | 1 | 2 (18) | 3 |
| Reflux gastritis | 1 (17) | 1 | 0 | 0 | 1 (9) | 1 |
| Regurgitation of food | 1 (17) | 1 | 0 | 0 | 1 (9) | 1 |
| Toothache | 1 (17) | 1 | 0 | 0 | 1 (9) | 1 |
| Skin and subcutaneous tissue disorders | 2 (33) | 26 | 3 (60) | 22 | 5 (45) | 48 |
| Rash | 2 (33) | 9 | 3 (60) | 6 | 5 (45) | 15 |
| Urticaria | 1 (17) | 13 | 2 (40) | 11 | 3 (27) | 24 |
| Swelling face | 1 (17) | 4 | 1 (20) | 1 | 2 (18) | 5 |
| Urticaria generalized | 0 | 0 | 2 (40) | 4 | 2 (18) | 4 |
| Musculoskeletal and connective tissue disorders | 0 | 0 | 1 (20) | 1 | 1 (9) | 1 |
| Muscle spasms | 0 | 0 | 1 (20) | 1 | 1 (9) | 1 |
| Reproductive system and breast disorders | 0 | 0 | 1 (20) | 1 | 1 (9) | 1 |
| Phimosis | 0 | 0 | 1 (20) | 1 | 1 (9) | 1 |
| General disorders and administration-site conditions | 5 (83) | 17 | 5 (100) | 7 | 10 (91) | 24 |
| Pyrexia | 5 (83) | 17 | 4 (80) | 4 | 9 (82) | 21 |
| Adverse drug reaction | 0 | 0 | 1 (20) | 1 | 1 (9) | 1 |
| Disease progression | 0 | 0 | 1 (20) | 1 | 1 (9) | 1 |
| Generalized edema | 0 | 0 | 1 (20) | 1 | 1 (9) | 1 |
| Investigations | 3 (50) | 5 | 1 (20) | 1 | 4 (36) | 6 |
| Blood iron increased | 1 (17) | 1 | 1 (20) | 1 | 2 (18) | 2 |
| Blood alkaline phosphatase increased | 1 (17) | 1 | 0 | 0 | 1 (9) | 1 |
| Heart rate increased | 1 (17) | 1 | 0 | 0 | 1 (9) | 1 |
| Platelet count increased | 1 (17) | 1 | 0 | 0 | 1 (9) | 1 |
| Weight decreased | 1 (17) | 1 | 0 | 0 | 1 (9) | 1 |
| Injury, poisoning and procedural complications | 1 (17) | 1 | 2 (40) | 3 | 3 (27) | 4 |
| Feeding tube complication | 1 (17) | 1 | 1 (20) | 2 | 2 (18) | 3 |
| Joint dislocation | 0 | 0 | 1 (20) | 1 | 1 (9) | 1 |

^a^Number (%) of patients in treatment group having the event.

^b^Number of events. Note that a patient may experience more than one event.

IV = intravenous; rhASA = recombinant human arylsulfatase A.
